# (*E*)-*N*-(3,3-Diphenyl­allyl­idene)-4-nitro­aniline

**DOI:** 10.1107/S1600536812040391

**Published:** 2012-09-29

**Authors:** Yong Koo Kang, Yong Seo Cho, Jae Kyun Lee, Byung-Yong Yu, Joo Hwan Cha

**Affiliations:** a Center for Neuro-Medicine, Korea Institute of Science & Technology, Hwarangro 14-gil, Seongbuk-gu, Seoul 136-791, Republic of Korea; b Advanced Analysis Center, Korea Institute of Science & Technology, Hwarangro 14-gil, Seongbuk-gu, Seoul 136-791, Republic of Korea

## Abstract

In the title compound, C_21_H_16_N_2_O_2_, the dihedral angles between the mean planes of the 4-nitro­phenyl ring and the two phenyl rings are 57.3 (5) and 16.8 (6)°. The imine group displays a C—C—N—C torsion angle of −24.9 (3)°.

## Related literature

For the structure of (*E*)-*N*-(3,3-diphenylallylidene)-3-nitroaniline, see: Cha *et al.* (2012 ▶). For other related structures, see: Khalaji *et al.* (2008 ▶); Khalaji & Harrison (2008 ▶).
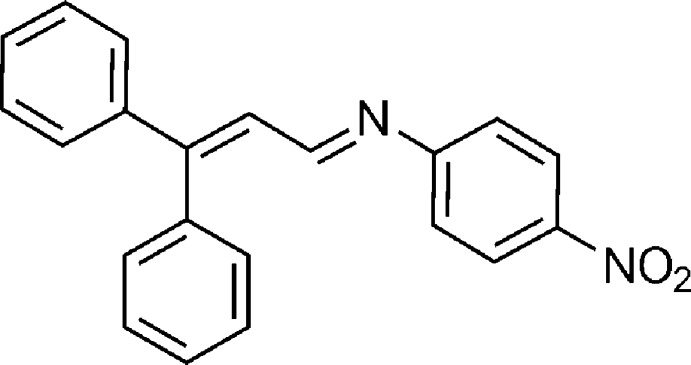


## Experimental

### 

#### Crystal data

C_21_H_16_N_2_O_2_
*M* *_r_* = 328.37
Monoclinic, 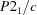

*a* = 9.4399 (8) Å
*b* = 23.1526 (16) Å
*c* = 8.1388 (5) Å
β = 108.039 (2)°
*V* = 1691.4 (3) Å^3^
*Z* = 4
Mo *K*α radiation
μ = 0.08 mm^−1^
*T* = 296 K
0.20 × 0.05 × 0.02 mm

#### Data collection

Rigaku R-AXIS RAPID diffractometer
Absorption correction: multi-scan (*ABSCOR*; Rigaku, 1995 ▶) *T*_min_ = 0.810, *T*_max_ = 0.998
16477 measured reflections
3864 independent reflections
2060 reflections with *F* ^2^ > 2σ(*F* ^2^)
*R*_int_ = 0.034

#### Refinement

*R*[*F* ^2^ > 2σ(*F* ^2^)] = 0.038
*wR*(*F* ^2^) = 0.113
*S* = 1.00
3864 reflections
234 parameters
H atoms treated by a mixture of independent and constrained refinement
Δρ_max_ = 0.14 e Å^−3^
Δρ_min_ = −0.18 e Å^−3^

### 

Data collection: *RAPID-AUTO* (Rigaku, 2006 ▶); cell refinement: *RAPID-AUTO*; data reduction: *RAPID-AUTO*; program(s) used to solve structure: *Il Milione* (Burla *et al.*, 2007 ▶); program(s) used to refine structure: *SHELXL97* (Sheldrick, 2008 ▶); molecular graphics: *CrystalStructure* (Rigaku, 2010 ▶); software used to prepare material for publication: *CrystalStructure*.

## Supplementary Material

Crystal structure: contains datablock(s) global, I. DOI: 10.1107/S1600536812040391/ff2082sup1.cif

Structure factors: contains datablock(s) I. DOI: 10.1107/S1600536812040391/ff2082Isup2.hkl

Supplementary material file. DOI: 10.1107/S1600536812040391/ff2082Isup3.cml

Additional supplementary materials:  crystallographic information; 3D view; checkCIF report

## Figures and Tables

**Fig. 1.**
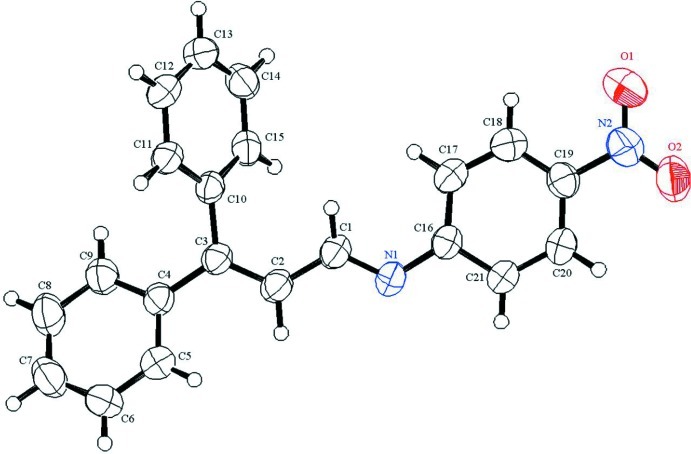
The molecular structure of the title compound showing the atomic numbering and 50% probability displacement ellipsoid.

## supplementary crystallographic information

### Comment

For the title compound (Fig. 1), C_21_H_16_N_2_O_2_, similar structures were described previously by (Khalaji *et al.*, 2008; Khalaji & Harrison, 2008). The dihedral angles between the mean planes of the central 4-nitrophenyl ring and the two benzene groups are (C10/C11/C12/C13/C14/C15) 57.33 (50)° and (C4/C5/C6/C7/C8/C9) 16.80 (55)°, respectively. The bond lengths and angles in the title molecule are unexceptional. The imine group displays a torsion angle [C17–C16–N1–C1 = -24.87 (23)°]. The plane of the nitro group is twisted by 1.86 (23)° out of the parent benzene ring plane.

### Experimental

To a solution of 4-nitroaniline (4.0 mmol) in ethanol (10 ml) was treated with equimolar quantities of substituted 2-phenylcinnamaldehydes. The mixture was refluxed for 5 h, and the progress of reaction was monitored by TLC. Upon completion,the solvent was removed under reduced pressure. The residue was purified by flash column chromatography to afford the title compound in 74% yield. Recrystallization from ethanol gave crystals suitable for X-ray analysis.

### Refinement

All hydrogen atoms were positioned geometrically and refined using a riding model with C—H = 0.93–0.99 Å and *U*iso(H) = 1.2 or 1.5 *U*eq(C).

### Crystal data

**Table:**

C_21_H_16_N_2_O_2_	*F*(000) = 688.00
*M**_r_* = 328.37	*D*_x_ = 1.289 Mg m^−^^3^
Monoclinic, *P*2_1_/*c*	Mo *K*α radiation, λ = 0.71075 Å
Hall symbol: -P 2ybc	Cell parameters from 9450 reflections
*a* = 9.4399 (8) Å	θ = 3.0–27.4°
*b* = 23.1526 (16) Å	µ = 0.08 mm^−^^1^
*c* = 8.1388 (5) Å	*T* = 296 K
β = 108.039 (2)°	Platelet, colourless
*V* = 1691.4 (3) Å^3^	0.20 × 0.05 × 0.02 mm
*Z* = 4	

### Data collection

**Table:**

Rigaku R-AXIS RAPID diffractometer	2060 reflections with *F*^2^ > 2σ(*F*^2^)
Detector resolution: 10.000 pixels mm^-1^	*R*_int_ = 0.034
ω scans	θ_max_ = 27.5°
Absorption correction: multi-scan (*ABSCOR*; Rigaku, 1995)	*h* = −12→12
*T*_min_ = 0.810, *T*_max_ = 0.998	*k* = −29→30
16477 measured reflections	*l* = −9→10
3864 independent reflections	

### Refinement

**Table:**

Refinement on *F*^2^	Secondary atom site location: difference Fourier map
*R*[*F*^2^ > 2σ(*F*^2^)] = 0.038	Hydrogen site location: inferred from neighbouring sites
*wR*(*F*^2^) = 0.113	H atoms treated by a mixture of independent and constrained refinement
*S* = 1.00	*w* = 1/[σ^2^(*F*_o_^2^) + (0.0576*P*)^2^] where *P* = (*F*_o_^2^ + 2*F*_c_^2^)/3
3864 reflections	(Δ/σ)_max_ < 0.001
234 parameters	Δρ_max_ = 0.14 e Å^−^^3^
0 restraints	Δρ_min_ = −0.18 e Å^−^^3^
Primary atom site location: structure-invariant direct methods	

### Special details

**Table:**

Geometry. All e.s.d.'s (except the e.s.d. in the dihedral angle between two l.s. planes) are estimated using the full covariance matrix. The cell e.s.d.'s are taken into account individually in the estimation of e.s.d.'s in distances, angles and torsion angles; correlations between e.s.d.'s in cell parameters are only used when they are defined by crystal symmetry. An approximate (isotropic) treatment of cell e.s.d.'s is used for estimating e.s.d.'s involving l.s. planes.
Refinement. Refinement was performed using all reflections. The weighted *R*-factor (*wR*) and goodness of fit (*S*) are based on *F*^2^. *R*-factor (gt) are based on *F*. The threshold expression of *F*^2^ > 2.0 σ(*F*^2^) is used only for calculating *R*-factor (gt).

### Fractional atomic coordinates and isotropic or equivalent isotropic displacement parameters (Å^2^)

**Table:**

	*x*	*y*	*z*	*U*_iso_*/*U*_eq_	
O1	−0.06733 (17)	0.42186 (6)	−0.26289 (19)	0.0940 (5)	
O2	0.04148 (15)	0.33937 (5)	−0.24984 (17)	0.0825 (4)	
N1	0.55099 (15)	0.47570 (4)	0.29682 (15)	0.0525 (4)	
N2	0.04088 (18)	0.38989 (6)	−0.20661 (18)	0.0655 (4)	
C1	0.57956 (19)	0.52982 (6)	0.32064 (19)	0.0497 (4)	
C2	0.70646 (19)	0.55014 (6)	0.45689 (19)	0.0517 (4)	
C3	0.73343 (17)	0.60637 (5)	0.50580 (17)	0.0446 (4)	
C4	0.87658 (17)	0.62390 (5)	0.63168 (18)	0.0466 (4)	
C5	1.0066 (2)	0.59193 (6)	0.6530 (3)	0.0612 (5)	
C6	1.1393 (2)	0.60752 (7)	0.7732 (3)	0.0743 (6)	
C7	1.1473 (3)	0.65514 (8)	0.8747 (3)	0.0739 (6)	
C8	1.0219 (3)	0.68818 (7)	0.8554 (3)	0.0711 (5)	
C9	0.88849 (19)	0.67281 (6)	0.7344 (2)	0.0587 (5)	
C10	0.61651 (17)	0.65090 (5)	0.43491 (17)	0.0443 (4)	
C11	0.64989 (18)	0.70200 (5)	0.36279 (18)	0.0506 (4)	
C12	0.5392 (2)	0.74154 (6)	0.2873 (2)	0.0585 (5)	
C13	0.3953 (2)	0.73211 (6)	0.2866 (2)	0.0622 (5)	
C14	0.36038 (19)	0.68283 (6)	0.3623 (2)	0.0605 (5)	
C15	0.47054 (18)	0.64259 (6)	0.43519 (18)	0.0508 (4)	
C16	0.42297 (18)	0.45804 (5)	0.16448 (19)	0.0485 (4)	
C17	0.2962 (2)	0.49148 (6)	0.0971 (3)	0.0621 (5)	
C18	0.1729 (2)	0.46956 (6)	−0.0263 (3)	0.0635 (5)	
C19	0.17478 (18)	0.41347 (6)	−0.08041 (19)	0.0519 (4)	
C20	0.29728 (19)	0.37881 (6)	−0.01558 (19)	0.0543 (4)	
C21	0.42090 (19)	0.40124 (6)	0.10584 (19)	0.0529 (4)	
H5	1.0034	0.5595	0.5847	0.0734*	
H6	1.2243	0.5854	0.7853	0.0891*	
H7	1.2369	0.6652	0.9565	0.0886*	
H8	1.0269	0.7208	0.9236	0.0853*	
H9	0.8048	0.6957	0.7214	0.0704*	
H11	0.7474	0.7094	0.3657	0.0607*	
H12	0.5622	0.7748	0.2367	0.0701*	
H13	0.3213	0.7589	0.2353	0.0746*	
H14	0.2635	0.6767	0.3641	0.0726*	
H15	0.4465	0.6094	0.4853	0.0610*	
H17	0.2945	0.5292	0.1358	0.0746*	
H18	0.0892	0.4925	−0.0726	0.0762*	
H20	0.2968	0.3408	−0.0530	0.0652*	
H21	0.5047	0.3781	0.1497	0.0634*	
H1	0.5198 (19)	0.5588 (6)	0.242 (2)	0.061 (5)*	
H2	0.7792 (18)	0.5202 (7)	0.518 (2)	0.061 (5)*	

### Atomic displacement parameters (Å^2^)

**Table:**

	*U*^11^	*U*^22^	*U*^33^	*U*^12^	*U*^13^	*U*^23^
O1	0.0638 (11)	0.1016 (10)	0.1000 (11)	0.0074 (8)	0.0011 (9)	−0.0062 (8)
O2	0.0716 (10)	0.0797 (8)	0.0925 (10)	−0.0162 (7)	0.0200 (8)	−0.0277 (7)
N1	0.0581 (10)	0.0394 (6)	0.0575 (8)	−0.0036 (6)	0.0143 (7)	−0.0043 (6)
N2	0.0580 (11)	0.0739 (10)	0.0646 (9)	−0.0055 (8)	0.0189 (8)	−0.0043 (8)
C1	0.0615 (11)	0.0387 (7)	0.0483 (9)	−0.0014 (7)	0.0162 (8)	−0.0004 (7)
C2	0.0607 (12)	0.0405 (8)	0.0510 (9)	0.0008 (7)	0.0131 (9)	0.0018 (7)
C3	0.0495 (10)	0.0398 (7)	0.0445 (8)	−0.0006 (7)	0.0144 (8)	0.0017 (6)
C4	0.0487 (10)	0.0424 (7)	0.0478 (8)	−0.0026 (7)	0.0134 (8)	0.0024 (7)
C5	0.0589 (13)	0.0495 (8)	0.0696 (11)	0.0047 (8)	0.0120 (9)	−0.0019 (8)
C6	0.0505 (13)	0.0662 (11)	0.0931 (14)	0.0038 (9)	0.0032 (11)	0.0073 (10)
C7	0.0600 (14)	0.0788 (12)	0.0671 (12)	−0.0143 (10)	−0.0031 (10)	0.0037 (10)
C8	0.0695 (14)	0.0710 (11)	0.0664 (11)	−0.0137 (10)	0.0119 (10)	−0.0176 (9)
C9	0.0543 (12)	0.0585 (9)	0.0609 (10)	−0.0034 (8)	0.0143 (9)	−0.0116 (8)
C10	0.0494 (10)	0.0392 (7)	0.0430 (8)	−0.0027 (6)	0.0121 (7)	−0.0053 (6)
C11	0.0503 (11)	0.0412 (7)	0.0606 (9)	−0.0009 (7)	0.0176 (8)	0.0004 (7)
C12	0.0640 (13)	0.0420 (8)	0.0680 (11)	0.0022 (8)	0.0185 (9)	0.0048 (7)
C13	0.0598 (13)	0.0530 (9)	0.0650 (10)	0.0094 (8)	0.0068 (9)	−0.0038 (8)
C14	0.0448 (11)	0.0663 (10)	0.0654 (10)	−0.0028 (8)	0.0096 (8)	−0.0106 (9)
C15	0.0508 (11)	0.0468 (8)	0.0528 (9)	−0.0092 (7)	0.0130 (8)	−0.0047 (7)
C16	0.0539 (11)	0.0413 (7)	0.0511 (9)	−0.0028 (7)	0.0174 (8)	0.0007 (7)
C17	0.0639 (13)	0.0422 (8)	0.0782 (12)	0.0032 (8)	0.0189 (10)	−0.0061 (8)
C18	0.0566 (12)	0.0553 (9)	0.0756 (11)	0.0060 (8)	0.0162 (10)	0.0015 (9)
C19	0.0503 (11)	0.0562 (9)	0.0499 (9)	−0.0061 (8)	0.0163 (8)	0.0001 (7)
C20	0.0615 (12)	0.0459 (8)	0.0556 (9)	−0.0049 (8)	0.0182 (9)	−0.0080 (7)
C21	0.0558 (11)	0.0410 (7)	0.0597 (10)	0.0001 (7)	0.0148 (9)	−0.0023 (7)

### Geometric parameters (Å, º)

**Table:**

O1—N2	1.229 (2)	C16—C21	1.3971 (19)
O2—N2	1.2220 (19)	C17—C18	1.377 (3)
N1—C1	1.2837 (17)	C18—C19	1.373 (2)
N1—C16	1.4077 (18)	C19—C20	1.372 (3)
N2—C19	1.465 (2)	C20—C21	1.376 (2)
C1—C2	1.436 (2)	C1—H1	0.978 (14)
C2—C3	1.3621 (19)	C2—H2	0.994 (15)
C3—C4	1.4775 (19)	C5—H5	0.930
C3—C10	1.4898 (19)	C6—H6	0.930
C4—C5	1.397 (3)	C7—H7	0.930
C4—C9	1.391 (2)	C8—H8	0.930
C5—C6	1.378 (3)	C9—H9	0.930
C6—C7	1.366 (3)	C11—H11	0.930
C7—C8	1.377 (3)	C12—H12	0.930
C8—C9	1.383 (3)	C13—H13	0.930
C10—C11	1.3993 (19)	C14—H14	0.930
C10—C15	1.392 (3)	C15—H15	0.930
C11—C12	1.382 (2)	C17—H17	0.930
C12—C13	1.374 (3)	C18—H18	0.930
C13—C14	1.384 (3)	C20—H20	0.930
C14—C15	1.385 (2)	C21—H21	0.930
C16—C17	1.388 (3)		
			
O1···C18	2.714 (2)	C12···H9^x^	3.0850
O1···C20	3.550 (2)	C12···H20^xi^	3.2069
O2···C18	3.5407 (19)	C12···H20^i^	3.3914
O2···C20	2.7292 (19)	C12···H21^xi^	3.2508
C1···C10	2.9401 (19)	C13···H7^v^	3.0602
C1···C15	3.056 (3)	C13···H7^xii^	3.3287
C1···C17	2.870 (3)	C13···H12^ix^	3.5143
C1···C21	3.541 (2)	C13···H21^xi^	3.5054
C2···C5	2.951 (3)	C14···H7^v^	3.1694
C2···C15	3.054 (3)	C14···H12^ix^	3.2070
C4···C7	2.799 (3)	C14···H13^ix^	3.4454
C4···C11	3.1206 (18)	C15···H12^ix^	3.0192
C5···C8	2.748 (3)	C15···H21^iii^	3.3480
C6···C9	2.744 (3)	C16···H6^vii^	3.3805
C9···C10	2.9858 (19)	C16···H15^iii^	3.1475
C9···C11	3.234 (2)	C16···H1^i^	3.533 (18)
C10···C13	2.791 (2)	C17···H6^v^	3.2504
C11···C14	2.767 (3)	C17···H2^iii^	3.428 (18)
C12···C15	2.757 (3)	C18···H6^v^	3.2005
C16···C19	2.763 (2)	C18···H18^ii^	2.9617
C17···C20	2.766 (2)	C19···H1^i^	3.577 (19)
C18···C21	2.753 (3)	C20···H9^iii^	3.3265
O1···C5^i^	3.412 (3)	C20···H11^i^	3.4240
O1···C14^ii^	3.577 (3)	C20···H12^vi^	3.2938
O1···C17^ii^	3.510 (3)	C20···H1^i^	3.229 (18)
O2···C4^i^	3.531 (3)	C21···H6^vii^	3.2035
O2···C5^i^	3.547 (3)	C21···H12^vi^	3.1805
O2···C8^iii^	3.501 (3)	C21···H15^iii^	3.1815
O2···C11^i^	3.451 (3)	C21···H1^i^	3.186 (18)
N1···C15^iii^	3.5459 (19)	H5···O1^iv^	3.5562
N2···C5^i^	3.546 (3)	H5···O1^i^	2.8966
N2···C8^iii^	3.583 (3)	H5···O2^i^	3.5184
C4···O2^i^	3.531 (3)	H5···N2^i^	3.1974
C5···O1^i^	3.412 (3)	H5···H5^vii^	3.0711
C5···O2^i^	3.547 (3)	H5···H18^iv^	3.0720
C5···N2^i^	3.546 (3)	H5···H2^vii^	3.0636
C6···C18^iv^	3.556 (3)	H6···N1^vii^	2.7984
C8···O2^iii^	3.501 (3)	H6···C1^vii^	3.5049
C8···N2^iii^	3.583 (3)	H6···C16^vii^	3.3805
C9···C20^iii^	3.496 (3)	H6···C17^iv^	3.2504
C11···O2^i^	3.451 (3)	H6···C18^iv^	3.2005
C11···C20^i^	3.551 (3)	H6···C21^vii^	3.2035
C14···O1^ii^	3.577 (3)	H6···H17^iv^	3.0168
C15···N1^iii^	3.5459 (19)	H6···H18^iv^	2.9159
C17···O1^ii^	3.510 (3)	H6···H21^vii^	2.5881
C18···C6^v^	3.556 (3)	H6···H2^vii^	3.4711
C20···C9^iii^	3.496 (3)	H7···C13^iv^	3.0602
C20···C11^i^	3.551 (3)	H7···C13^viii^	3.3287
O1···H18	2.4172	H7···C14^iv^	3.1694
O2···H20	2.4465	H7···H13^iv^	3.0603
N1···H17	2.6737	H7···H13^viii^	2.8029
N1···H21	2.5303	H7···H14^iv^	3.2592
N1···H2	2.560 (15)	H7···H17^iv^	3.4423
N2···H18	2.5958	H7···H21^vii^	3.0016
N2···H20	2.6112	H8···O2^xi^	3.0638
C1···H15	2.7947	H8···O2^iii^	3.2352
C1···H17	2.6454	H8···H11^ix^	3.0030
C2···H5	2.6798	H8···H13^iv^	3.2512
C2···H15	2.8825	H8···H13^viii^	3.5967
C3···H5	2.6597	H8···H14^viii^	3.3943
C3···H9	2.6611	H9···C11^ix^	3.1798
C3···H11	2.6654	H9···C12^ix^	3.0850
C3···H15	2.6624	H9···C20^iii^	3.3265
C3···H1	2.686 (14)	H9···H11^ix^	2.6245
C4···H6	3.2537	H9···H12^ix^	2.4291
C4···H8	3.2566	H9···H20^iii^	3.2436
C4···H11	2.9085	H11···O2^i^	2.7014
C4···H2	2.632 (15)	H11···N2^i^	3.5436
C5···H7	3.2220	H11···C8^x^	3.5317
C5···H9	3.2201	H11···C9^x^	3.3510
C5···H2	2.665 (15)	H11···C20^i^	3.4240
C6···H8	3.2111	H11···H8^x^	3.0030
C7···H5	3.2152	H11···H9^x^	2.6245
C7···H9	3.2237	H11···H20^xi^	3.4859
C8···H6	3.2106	H11···H20^i^	2.7103
C9···H5	3.2198	H12···C9^x^	3.3156
C9···H7	3.2303	H12···C10^x^	3.1679
C9···H11	3.0040	H12···C11^x^	3.4341
C10···H9	2.6659	H12···C13^x^	3.5143
C10···H12	3.2524	H12···C14^x^	3.2070
C10···H14	3.2566	H12···C15^x^	3.0192
C10···H1	2.639 (14)	H12···C20^xi^	3.2938
C10···H2	3.365 (15)	H12···C21^xi^	3.1805
C11···H9	2.8348	H12···H9^x^	2.4291
C11···H13	3.2307	H12···H15^x^	3.3446
C11···H15	3.2342	H12···H20^xi^	2.9354
C11···H1	3.567 (14)	H12···H20^i^	3.5216
C12···H14	3.2279	H12···H21^xi^	2.7100
C13···H11	3.2276	H13···C6^xii^	3.5979
C13···H15	3.2301	H13···C7^xii^	3.0178
C14···H12	3.2269	H13···C8^xii^	3.4871
C14···H1	3.519 (16)	H13···C14^x^	3.4454
C15···H9	3.5134	H13···H7^v^	3.0603
C15···H11	3.2347	H13···H7^xii^	2.8029
C15···H13	3.2339	H13···H8^v^	3.2512
C15···H1	2.629 (16)	H13···H8^xii^	3.5967
C16···H18	3.2440	H13···H14^x^	3.2598
C16···H20	3.2571	H13···H21^xi^	3.2012
C16···H1	2.513 (14)	H14···O1^ii^	2.8941
C17···H21	3.2283	H14···O2^ii^	2.7632
C17···H1	2.594 (15)	H14···N2^ii^	3.1621
C18···H20	3.2333	H14···H7^v^	3.2592
C19···H17	3.2126	H14···H8^xii^	3.3943
C19···H21	3.2039	H14···H13^ix^	3.2598
C20···H18	3.2316	H15···O1^ii^	3.5497
C21···H17	3.2311	H15···N1^iii^	2.6463
H5···H6	2.2948	H15···C16^iii^	3.1475
H5···H2	2.2107	H15···C21^iii^	3.1815
H6···H7	2.2940	H15···H12^ix^	3.3446
H7···H8	2.3093	H15···H21^iii^	2.8763
H8···H9	2.3029	H17···O1^ii^	2.8853
H9···H11	2.7942	H17···C6^v^	3.3861
H11···H12	2.3046	H17···H6^v^	3.0168
H12···H13	2.3006	H17···H7^v^	3.4423
H13···H14	2.3162	H17···H18^ii^	3.5327
H14···H15	2.3093	H17···H2^iii^	3.3055
H15···H17	3.3272	H18···O1^ii^	3.4312
H15···H1	2.5748	H18···C5^v^	3.1325
H17···H18	2.3059	H18···C6^v^	3.0420
H17···H1	2.1447	H18···C18^ii^	2.9617
H20···H21	2.3054	H18···H5^v^	3.0720
H1···H2	2.91 (2)	H18···H6^v^	2.9159
O1···H5^v^	3.5562	H18···H17^ii^	3.5327
O1···H5^i^	2.8966	H18···H18^ii^	2.3625
O1···H14^ii^	2.8941	H20···C9^iii^	3.5624
O1···H15^ii^	3.5497	H20···C10^i^	3.4559
O1···H17^ii^	2.8853	H20···C11^vi^	3.5356
O1···H18^ii^	3.4312	H20···C11^i^	2.8948
O1···H2^v^	2.976 (15)	H20···C12^vi^	3.2069
O2···H5^i^	3.5184	H20···C12^i^	3.3914
O2···H8^vi^	3.0638	H20···H9^iii^	3.2436
O2···H8^iii^	3.2352	H20···H11^vi^	3.4859
O2···H11^i^	2.7014	H20···H11^i^	2.7103
O2···H14^ii^	2.7632	H20···H12^vi^	2.9354
N1···H6^vii^	2.7984	H20···H12^i^	3.5216
N1···H15^iii^	2.6463	H20···H1^i^	3.5219
N2···H5^i^	3.1974	H21···C6^vii^	3.2386
N2···H11^i^	3.5436	H21···C7^vii^	3.4398
N2···H14^ii^	3.1621	H21···C12^vi^	3.2508
C1···H6^vii^	3.5049	H21···C13^vi^	3.5054
C5···H18^iv^	3.1325	H21···C15^iii^	3.3480
C6···H13^viii^	3.5979	H21···H6^vii^	2.5881
C6···H17^iv^	3.3861	H21···H7^vii^	3.0016
C6···H18^iv^	3.0420	H21···H12^vi^	2.7100
C6···H21^vii^	3.2386	H21···H13^vi^	3.2012
C7···H13^viii^	3.0178	H21···H15^iii^	2.8763
C7···H21^vii^	3.4398	H21···H1^i^	3.4464
C8···H11^ix^	3.5317	H1···C16^i^	3.533 (18)
C8···H13^viii^	3.4871	H1···C19^i^	3.577 (19)
C9···H11^ix^	3.3510	H1···C20^i^	3.229 (18)
C9···H12^ix^	3.3156	H1···C21^i^	3.186 (18)
C9···H20^iii^	3.5624	H1···H20^i^	3.5219
C10···H12^ix^	3.1679	H1···H21^i^	3.4464
C10···H20^i^	3.4559	H2···O1^iv^	2.976 (15)
C11···H9^x^	3.1798	H2···C17^iii^	3.428 (18)
C11···H12^ix^	3.4341	H2···H5^vii^	3.0636
C11···H20^xi^	3.5356	H2···H6^vii^	3.4711
C11···H20^i^	2.8948	H2···H17^iii^	3.3055
			
C1—N1—C16	119.31 (11)	C16—C21—C20	121.30 (14)
O1—N2—O2	123.12 (15)	N1—C1—H1	121.2 (9)
O1—N2—C19	118.28 (14)	C2—C1—H1	117.1 (9)
O2—N2—C19	118.59 (14)	C1—C2—H2	116.2 (9)
N1—C1—C2	121.48 (13)	C3—C2—H2	118.7 (9)
C1—C2—C3	125.06 (13)	C4—C5—H5	119.325
C2—C3—C4	120.88 (12)	C6—C5—H5	119.324
C2—C3—C10	119.95 (12)	C5—C6—H6	119.734
C4—C3—C10	119.16 (11)	C7—C6—H6	119.722
C3—C4—C5	121.35 (12)	C6—C7—H7	120.152
C3—C4—C9	121.78 (14)	C8—C7—H7	120.151
C5—C4—C9	116.88 (13)	C7—C8—H8	120.032
C4—C5—C6	121.35 (15)	C9—C8—H8	120.026
C5—C6—C7	120.54 (18)	C4—C9—H9	119.220
C6—C7—C8	119.70 (16)	C8—C9—H9	119.214
C7—C8—C9	119.94 (16)	C10—C11—H11	119.704
C4—C9—C8	121.57 (16)	C12—C11—H11	119.705
C3—C10—C11	120.76 (15)	C11—C12—H12	119.760
C3—C10—C15	121.25 (13)	C13—C12—H12	119.762
C11—C10—C15	117.98 (13)	C12—C13—H13	119.990
C10—C11—C12	120.59 (16)	C14—C13—H13	119.985
C11—C12—C13	120.48 (15)	C13—C14—H14	120.184
C12—C13—C14	120.02 (15)	C15—C14—H14	120.177
C13—C14—C15	119.64 (17)	C10—C15—H15	119.387
C10—C15—C14	121.23 (14)	C14—C15—H15	119.387
N1—C16—C17	124.83 (12)	C16—C17—H17	119.558
N1—C16—C21	116.92 (13)	C18—C17—H17	119.570
C17—C16—C21	118.10 (13)	C17—C18—H18	120.307
C16—C17—C18	120.87 (14)	C19—C18—H18	120.326
C17—C18—C19	119.37 (15)	C19—C20—H20	120.599
N2—C19—C18	118.89 (14)	C21—C20—H20	120.595
N2—C19—C20	119.55 (13)	C16—C21—H21	119.339
C18—C19—C20	121.53 (14)	C20—C21—H21	119.357
C19—C20—C21	118.81 (14)		
			
C1—N1—C16—C17	−24.9 (3)	C4—C5—C6—C7	−0.3 (3)
C1—N1—C16—C21	159.61 (15)	C5—C6—C7—C8	−0.7 (3)
C16—N1—C1—C2	178.26 (15)	C6—C7—C8—C9	0.5 (3)
O1—N2—C19—C18	1.9 (3)	C7—C8—C9—C4	0.8 (3)
O1—N2—C19—C20	−179.91 (16)	C3—C10—C11—C12	175.88 (11)
O2—N2—C19—C18	−177.54 (15)	C3—C10—C15—C14	−177.06 (11)
O2—N2—C19—C20	0.7 (3)	C11—C10—C15—C14	1.7 (2)
N1—C1—C2—C3	−170.53 (16)	C15—C10—C11—C12	−2.90 (19)
C1—C2—C3—C4	−171.88 (16)	C10—C11—C12—C13	2.0 (3)
C1—C2—C3—C10	9.4 (3)	C11—C12—C13—C14	0.2 (3)
C2—C3—C4—C5	25.9 (3)	C12—C13—C14—C15	−1.4 (3)
C2—C3—C4—C9	−154.08 (15)	C13—C14—C15—C10	0.4 (3)
C2—C3—C10—C11	−129.75 (15)	N1—C16—C17—C18	−176.61 (15)
C2—C3—C10—C15	49.0 (2)	N1—C16—C21—C20	176.00 (14)
C4—C3—C10—C11	51.5 (2)	C17—C16—C21—C20	0.2 (3)
C4—C3—C10—C15	−129.75 (14)	C21—C16—C17—C18	−1.1 (3)
C10—C3—C4—C5	−155.41 (13)	C16—C17—C18—C19	1.4 (3)
C10—C3—C4—C9	24.6 (3)	C17—C18—C19—N2	177.57 (16)
C3—C4—C5—C6	−178.48 (14)	C17—C18—C19—C20	−0.6 (3)
C3—C4—C9—C8	178.22 (13)	N2—C19—C20—C21	−178.51 (14)
C5—C4—C9—C8	−1.7 (3)	C18—C19—C20—C21	−0.3 (3)
C9—C4—C5—C6	1.5 (3)	C19—C20—C21—C16	0.5 (3)

Symmetry codes: (i) −*x*+1, −*y*+1, −*z*; (ii) −*x*, −*y*+1, −*z*; (iii) −*x*+1, −*y*+1, −*z*+1; (iv) *x*+1, *y*, *z*+1; (v) *x*−1, *y*, *z*−1; (vi) −*x*+1, *y*−1/2, −*z*+1/2; (vii) −*x*+2, −*y*+1, −*z*+1; (viii) *x*+1, −*y*+3/2, *z*+1/2; (ix) *x*, −*y*+3/2, *z*+1/2; (x) *x*, −*y*+3/2, *z*−1/2; (xi) −*x*+1, *y*+1/2, −*z*+1/2; (xii) *x*−1, −*y*+3/2, *z*−1/2.
